# Bimanual Motor Coordination in Older Adults Is Associated with Increased Functional Brain Connectivity – A Graph-Theoretical Analysis

**DOI:** 10.1371/journal.pone.0062133

**Published:** 2013-04-29

**Authors:** Marcus H. Heitger, Daniel J. Goble, Thijs Dhollander, Patrick Dupont, Karen Caeyenberghs, Alexander Leemans, Stefan Sunaert, Stephan P. Swinnen

**Affiliations:** 1 Motor Control Laboratory, Research Center for Movement Control and Neuroplasticity, Group Biomedical Sciences, KU Leuven, Leuven-Heverlee, Belgium; 2 Medical Imaging Research Center, University Hospital Gasthuisberg, KU Leuven, Leuven, Belgium; 3 Laboratory for Cognitive Neurology, Department of Neurosciences, Group Biomedical Sciences, KU Leuven, Leuven, Belgium; 4 Image Sciences Institute, University Medical Center Utrecht, Utrecht, The Netherlands; 5 Medical Imaging Research Center, Radiology, University Hospital Gasthuisberg KU Leuven, Leuven, Belgium; Beijing Normal University, Beijing, China

## Abstract

In bimanual coordination, older and younger adults activate a common cerebral network but the elderly also have additional activation in a secondary network of brain areas to master task performance. It remains unclear whether the functional connectivity within these primary and secondary motor networks differs between the old and the young and whether task difficulty modulates connectivity. We applied graph-theoretical network analysis (GTNA) to task-driven fMRI data in 16 elderly and 16 young participants using a bimanual coordination task including in-phase and anti-phase flexion/extension wrist movements. Network nodes for the GTNA comprised task-relevant brain areas as defined by fMRI activation foci. The elderly matched the motor performance of the young but showed an increased functional connectivity in both networks across a wide range of connectivity metrics, i.e., higher mean connectivity degree, connection strength, network density and efficiency, together with shorter mean communication path length between the network nodes and also a lower betweenness centrality. More difficult movements showed an increased connectivity in both groups. The network connectivity of both groups had “small world” character. The present findings indicate (a) that bimanual coordination in the aging brain is associated with a higher functional connectivity even between areas also activated in young adults, independently from task difficulty, and (b) that adequate motor coordination in the context of task-driven bimanual control in older adults may not be solely due to additional neural recruitment but also to aging-related changes of functional relationships between brain regions.

## Introduction

Aging adversely affects the quality of motor control, and it is commonly accepted that older adults will show poorer motor output with regard to speed, coordination of limb movement, or balance compared to younger adults [1], [2], [3], [4], [5], [6], [7]. However, despite the fact that normal aging is associated with a declining structural integrity of the brain [8], [9], [10], [11], [12], [13], [14], [15], [16], there is an increasing body of evidence from imaging studies suggesting that the aging brain can efficiently counteract the neurobiological changes via a compensatory reorganization of brain function, thus preserving performance levels comparable to those of young adults in many areas of cognition and motor control [1], [2], [17], [18], [19], [20], [21], [22], [23]. It has been shown that older adults commonly recruit a wider network of brain regions than younger adults during task performance [21], [24]. This increased activation has also been reported for a wide range of movement tasks including auditorily paced thumb to index finger tapping [25], finger abduction/adduction [26], wrist flexion/extension [26], sequential finger presses [1], [2], hand force production [27], [28], hand/foot coordination [17], [18], [29], and bimanual motor coordination [30].

Whilst network functionality of the brain is adversely affected by neurodegenerative diseases prevalent in older age groups, such as Alzheimer’s disease [31], [32], [33], [34], [35], it has been shown that normal aging is also associated with changes in network functionality [36], [37], [38], [39], [40]. A number of aging studies have focused on the assessment of functional brain connectivity in the context of resting state activations [36], [37], [38], [39], [40]. However, some studies have examined task-related brain connectivity: a study by Langan and colleagues [41] combined task-driven activation and resting state fMRI by using a joystick-driven motor paradigm to identify regions of interest for a resting state functional connectivity analysis. Their study demonstrated a higher resting state connectivity in the right hemisphere and stronger sensorimotor cortex interhemispheric connectivity in older subjects. Only one previous study has assessed aspects of the functional connectivity of the aging brain in a task-related motor context [42]. The study by Park et al. examined connectivity across a large number of brain regions by applying an atlas-based a-priori parcellation of the brain into 93 brain areas. However, whilst Park et al. could demonstrate increased network connectivity in parietal-occipital cerebellar related networks during dominant hand use in older subjects, their study focused on efficiency of information transfer as sole measure but provided no information on other aspects of functional connectivity associated with aging.

The present study aimed at expanding the limited previous work on the impact of aging on functional connectivity in the context of task-related motor control. This aim included the questions of whether (a) the greater neural activation of the older adults during complex bimanual coordination tasks may indeed be associated with an altered network functionality and (b) whether any disparities in network functionality between the young and old may be subject to the task difficulty associated with easier (intrinsic) in-phase (IP) versus more difficult anti-phase (AP) coordination modes. The present work built on the study by Goble et al. [30], who examined cerebral activations during a bimanual coordination task and were the first to demonstrate that bimanual movements require greater neural resources for old adults in order to match the level of performance seen in younger subjects.

The present work expands the initial analyses of Goble and colleagues [30] and was inspired by two main hypotheses: (a) based on previous studies of neural network functionality in aging populations in a non-motor context [37], [43], [44], we expected an effect of age with a decrease in functional connectivity in older adults within the ‘common’ motor-related brain network (i.e., the network consisting of regions similarly activated by the old and the young); (b) we expected an effect of task complexity whereby, compared to the easier IP movements, the performance of the more effortful AP task would be associated with an increased functional connectivity between brain regions. Furthermore, previous studies have reported positive correlations specifically in older test groups between motor performance and levels of brain activation in the areas with increased activation compared to young controls [18], [30]. Hence, we examined if such correlations between BOLD response and motor performance are mirrored in similar associations between measures of functional connectivity in the brain and motor output. We anticipated that this study would be able to help us better understand the adaptive processes that play a crucial role in preserving the functionality of the aging brain.

## Materials and Methods

### Participants

The present analysis is based on data acquired by the study of Goble et al. [30]. As part of that study, 16 (mean age = 68.3 years; range, 61.1–78.7 years; eight females) and 16 gender-matched young adults (mean age = 25.7 years; range, 21.0–30.9 years) were recruited from the local community. Participants were right-handed as indicated by laterality quotients of greater than +90 on the Edinburgh Handedness Inventory [45]. Subjects were free of neuromuscular impairment at the time of testing and were not under psychoactive or vasoactive medication. All subjects scored within normal limits (i.e., score >27) on the Mini-Mental State Examination [46]. Written informed consent was obtained for all participants and procedures were conducted following guidelines established by the ethics committee of Biomedical Research at the Katholieke Universiteit Leuven in compliance with Declaration of Helsinki. The study protocol and consent procedures were approved by the ethics committee of Biomedical Research at the Katholieke Universiteit Leuven.

### Task Description and Procedure

Subjects performed alternating 21-s blocks of three task conditions over four runs (i.e., time series) during functional magnetic resonance imaging (fMRI) [30]. In the first task condition, involving in-phase (IP) coordination, subjects made wrist flexion and extension movements in a mirror symmetric fashion with respect to body midline (i.e., with simultaneous activation of homologous muscles). The second task condition involved anti-phase (AP) coordination, with parallel motions of the hands via simultaneous flexion of one wrist and extension of the other, and then visa versa. In the third task, a rest condition was included where subjects abstained from moving either hand. The three tasks were performed in the presence of clearly audible pacing tones, which were used to set movement frequency for the IP and AP task conditions. Subjects were instructed to move smoothly and continuously, while timing each peak flexion/extension wrist movement with the occurrence of a tone. The frequency of tone presentation varied between blocks in a balanced fashion and corresponded to each subject’s relative capability in the AP coordination task: for each subject, task speeds were set at 45, 60, 75, and 90% of the subject’s “critical frequency”. This critical frequency was calculated as the maximum speed at which subjects were able to maintain AP task performance within ±45 deg of relative phase for at least 3 s. Importantly, this protocol lead to normalizing task difficulty (and thus effort) on an individual subject basis leading to group performances (measured by phase accuracy/stability) that were matched for young and old subjects. In turn, this ensured that any group differences in BOLD activation were unrelated to concomitant differences in participants’ ability to perform the study tasks, an important consideration because older adults show poorer performance particularly in unmatched bimanual tasks wherein the two hands are moving out of phase [3], [47]. Subcritical speeds were chosen to prevent phase transitions. Key measures of motor performance were phase accuracy (mean phase error in degrees) and phase stability (SD of relative phase). The mean phase error was determined as the average absolute deviation between the obtained relative phase and the target relative phase for in-phase (i.e., 0°) or antiphase (i.e., 180°) movements. The standard deviation of mean phase error was then quantified to provide a measure of coordination stability (i.e., phase variability) [30].

### MRI Scanning

Within 2 days before all scanning sessions, 45 min of practice was given in a mock scanner to familiarize subjects with task procedures and the scan environment. On the actual day of testing, subjects were placed head first and supine in the scanner with arms positioned along the trunk and elbows flexed at approximately 45 deg. This position was maintained throughout the scanning procedure with the aid of supportive cushioning. A bite-bar was used to minimize movements of the head, and a mirror was utilized to allow vision of images from an LCD projection system displayed on a screen mounted above the shoulders. This setup was used to cue the different task conditions during each scan run, preventing subjects from seeing their hands during the movement task. Subjects wore headphones for communication with the experimenter and for hearing auditory pacing tones. Orthoses, which limited wrist movement to flexion and extension, were attached to the left and right arms along the forearm and hand segments measuring joint displacement at a spatial resolution of 0.09 deg [30]. The displacement signal was available in real-time to the researchers during scanning, allowing them to ensure that subjects were complying with task instructions. This signal was also recorded for off-line analysis following the experimental session.

Image acquisition was achieved using a Siemens 3-T Magnetom Trio MRI scanner (Siemens, Erlangen, Germany) with standard head coil [30]. Each scanning session included a high-resolution T1-weighted image (MPRAGE; TR = 2,300 ms, echo time [TE] = 2.98 ms, 1×1×1.1 mm voxels, field of view: 240×256, 160 sagittal slices) for anatomical detail. Functional (fMRI) data were acquired over four time series (i.e., runs) with an interleaved EPI pulse sequence for T2*-weighted images (TR = 3,000 ms, TE = 30 ms, flip angle = 90°, 50 oblique slices each 2.8 mm thick, interslice gap 0.028 mm, in-plane resolution 2.5×2.5 mm, 80×80 matrix). Three “dummy” scans at the beginning of each run were discarded from analysis to allow for scanner equilibration. Each run lasted 378 s (6.3 min) consisting of six blocks of the three task conditions with each condition lasting 21 s (i.e., seven whole brain images). The order of conditions within a block was randomized across time series and the auditorily paced movement frequency for each block was randomized according to a balanced presentation across all blocks. Rest periods of approximately 3 min were inserted following each time series. Imaging data processing used Statistical Parametric Mapping (SPM) 5 software (Wellcome Department of Imaging Neuroscience, London, UK) implemented in Matlab 7.4 (MathWorks, Natick, MA).

### Neural Network Definition

**Figure 1:**
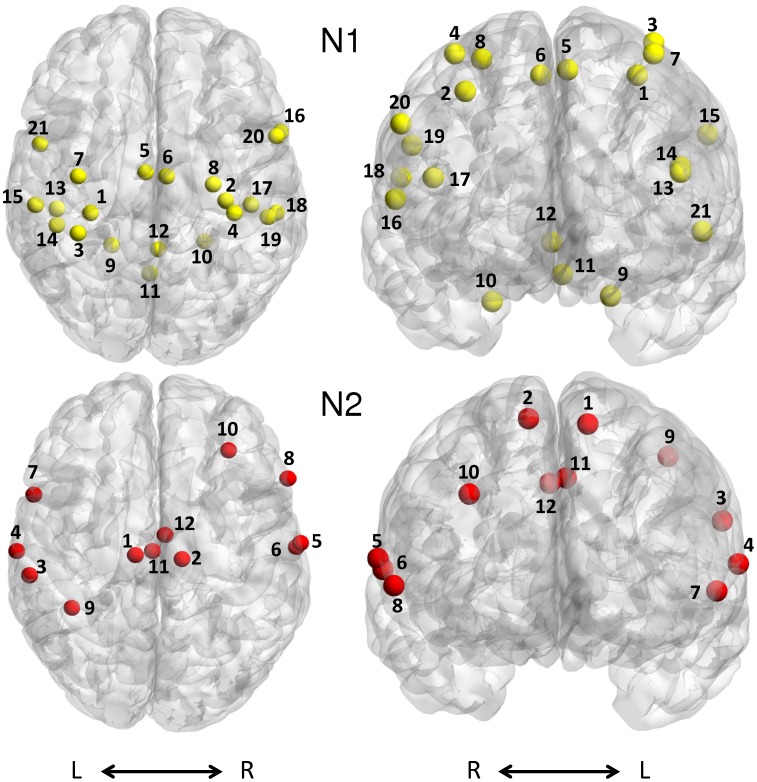
Neuroanatomical location of network nodes: N1 - brain regions with significant activation in both groups (i.e., brain regions representing common regions between young and old subjects), N2 - brain regions showing significantly higher activation (overactivation) in the old compared to the young group. Extent of network nodes in this figure is limited to 4 mm to visualize the precise node coordinates. Axial view: dorsal perspective; coronal view: anterior perspective.

**Figure 2:**
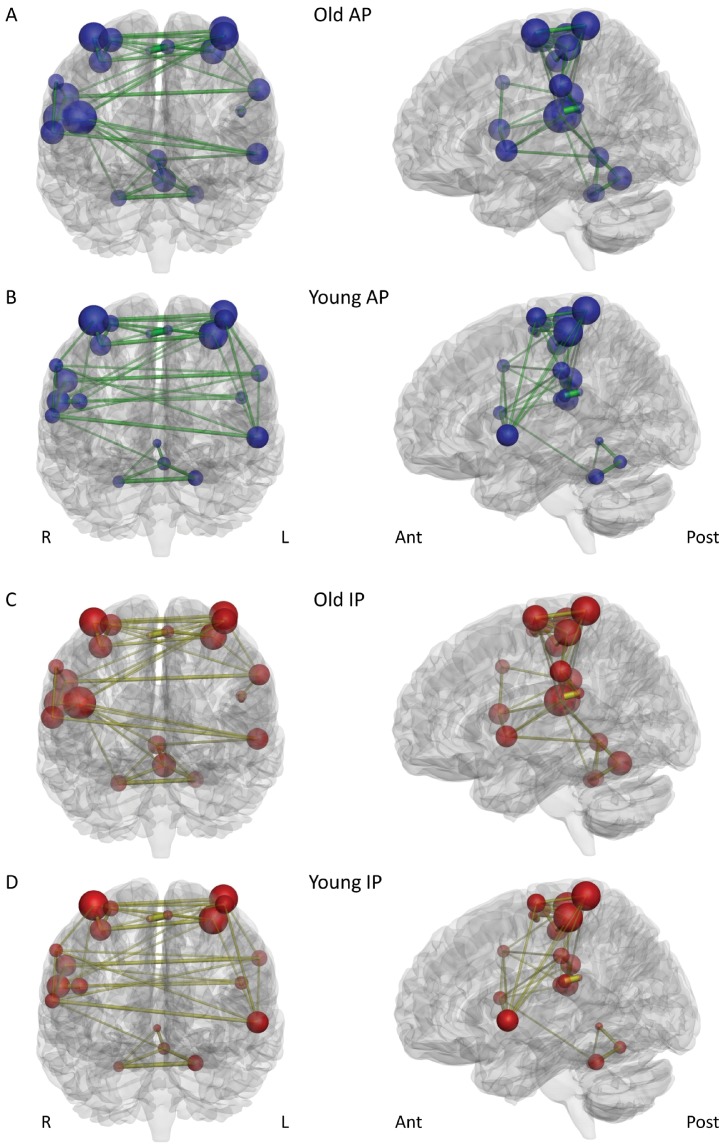
Visualization of key functional connectivity metrics in network N1 (i.e., the common network employed by both old and young participants) during AP movements (A+B), and during IP movements (C+D). Each brain region is represented by a sphere. Sphere size = mean connectivity degree; sphere transparency = mean betweenness centrality (network nodes which participate in many shortest paths, i.e., have a higher betweenness centrality, are less transparent than nodes with a low betweenness centrality). The edge width and transparency represent the summed strength of the partial correlations between nodes (i.e., the networks shown here were constructed by summing the partial correlation matrices of all participants in each group), edge width = increasing width represents stronger connections; edge transparency = less transparency indicates stronger connections. In order to visualize the main functional topology of the networks more clearly, only connections with a summed partial correlation strength of more than 1.0 are shown.

**Figure 3:**
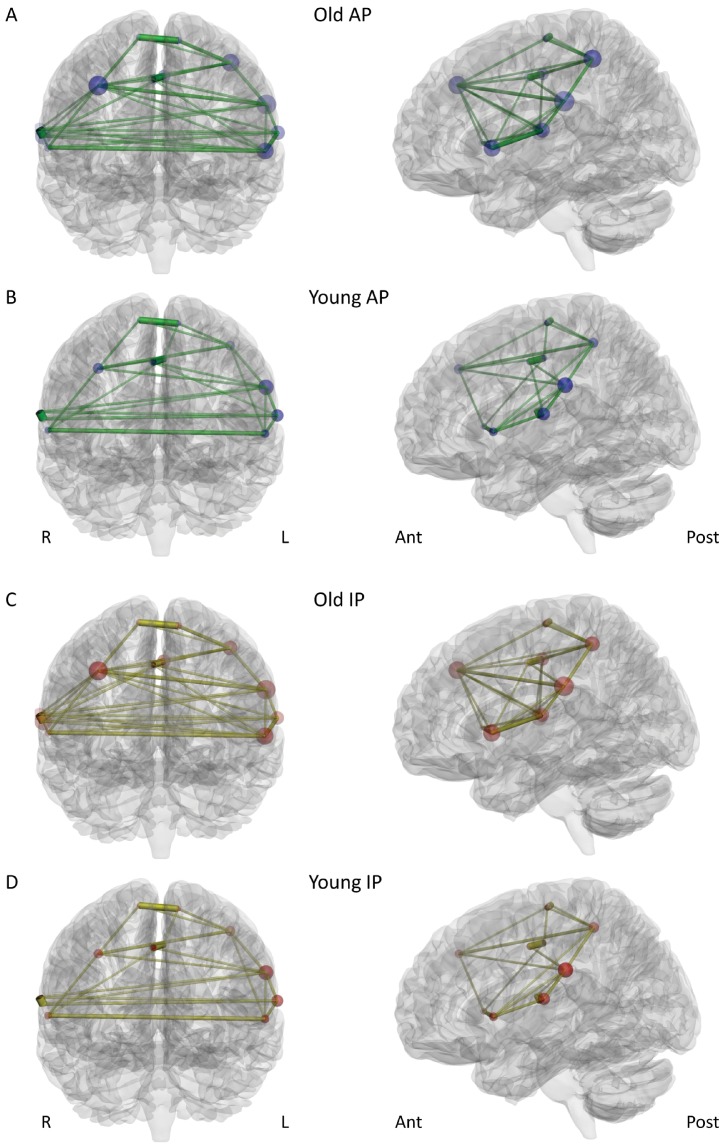
Visualization of key functional connectivity metrics in network N2 (i.e., the overactivation network of the older participants) during AP movements (A+B), and during IP movements (C+D). Figure properties as in Figure 2.

This study applied a data driven network definition, which was directly based on task-related cerebral activations in the subject groups as published in Goble et al. [30], comprising only brain areas active during performance of the present motor tasks [30]. This resulted in 2 cerebral networks to be examined via a graph-theoretical network analysis (GTNA): (1) brain regions with significant activation in both groups (i.e., brain regions representing common regions between young and old subjects; in each group, the activation of these areas compared to rest exceeded a FDR corrected threshold of *P*<0.01) [30] (network N1 including 21 ROIs, Table 1, Figures 1 and 2, for the extent of cortical activations and cluster sizes see Table 1 and Figure 3 in Goble et al., 2010) and (2) brain regions showing significantly higher activation (overactivation) in the old compared to the young group (network N2 with 12 ROIs, Table 2, Figures 1 and 3, for the extent of cortical activations and cluster sizes see Table 2 and Figure 3 in Goble et al., 2010). Network N1 included areas typically observed during motor coordination tasks, namely the sensorimotor cortices (SMI), supplementary motor area (SMA), cerebellum, and dorsal premotor cortices (PMd), as well as in the right ventral premotor cortex (PMv). Additionally, the network included areas in the left and right lateral fissures involving regions such as secondary somatosensory area (SII), primary auditory cortex (AI), and the IFG (pars opercularis). Network N2 comprised several areas that were significantly more active with age, including parts of the SMA and areas along the left and right lateral fissures (SII, IFG pars opercularis), as well as areas in bilateral middle cingulate cortex, secondary auditory area (AII), left inferior parietal cortex (IPC), and right DLPFC.

**Table 1:** Areas with significant activation in old and young individuals.

Activation peak location	Side (Fig. 1 label)	x	y	z
Precentral gyrus (MI, BA 4)	L (1)	−30	−30	58
	R (2)	34	−24	52
Postcentral gyrus (SI, BA 1/3)	L (3)	−36	−40	70
	R (4)	38	−30	66
Middle frontal gyrus (SMA, BA 6)	L (5)	−4	−10	60
	R (6)	6	−12	58
Precentral gyrus (PMd, BA 6)	L (7)	−36	−12	66
	R (8)	28	−16	64
Cerebellar hemisphere (IV–V)	L (9)	−20	−46	−24
	R (10)	24	−44	−26
Cerebellar vermis (IV–V)	L (11)	−2	−60	−16
	R (12)	2	−48	−4
Parietal operculum (SII, BA 43)	L (13)	−46	−28	22
Superior temporal gyrus (AI, BA 41)	L (14)	−46	−36	24
Supramarginal gyrus (SI, BA 2)	L (15)	−56	−26	36
IFG (pars opercularis, BA 44)	R (16)	60	10	12
Parietal operculum (SII, BA 43)	R (17)	46	−26	20
Superior temporal gyrus (AI, BA 41)	R (18)	58	−30	20
Supramarginal gyrus (SI, BA 2)	R (19)	54	−32	32
Precentral gyrus (PMv, BA 6)	R (20)	58	8	40
IFG (pars opercularis, BA 44)	L (21)	−54	4	0

**Table 2:** Areas with significant overactivation in old vs. young individuals.

Activation peak location	Side (Fig. 1 label)	x	y	z
Superior frontal gyrus (SMA, BA 6)	L (1)	−10	−18	64
	R (2)	12	−20	66
Parietal operculum (SII, BA43)	L (3)	−60	−28	28
Superior temporal gyrus (AII, BA 22)	L (4)	−66	−16	12
Parietal operculum (SII, BA 43)	R (5)	68	−12	14
Superior temporal gyrus (AII, BA 22)	R (6)	66	−14	10
IFG (pars opercularis, BA 44)	L (7)	−58	12	2
IFG (pars opercularis, BA 44)	R (8)	62	20	4
Inferior parietal cortex (BA 40)	L (9)	−40	−44	52
Middle frontal gyrus (DLPFC, BA 46)	R (10)	34	34	38
Middle cingulate cortex (BA 23)	L (11)	−2	−16	44
	R (12)	4	−8	42

The brain regions in each of these 2 networks were then defined as regions of interest (ROIs) representing the network components (nodes) for the GTNA (Figures 1–3). In each individual subject, separate analyses were conducted for each network. We chose to conduct separate analyses for the common and the overactivation networks because we considered that the effect of age might not equally manifest in the overactivation networks.

In each network, ROI size was defined by placing a sphere with radius 6 mm around the MNI coordinates of the ROI activation maximum. For each ROI in each subject, the BOLD average time series (AVT) were then extracted for the runs. The AVT extraction included whitening, filtering and removing null space of contrast using SPM code. During extraction, the time series was adjusted using the contrast ‘Task of interest [either AP or IP] – Rest’. Hence, this included separate extractions for the in-phase and the anti-phase epochs. The average time series was the average (across voxels in a ROI) of all voxel-based time series.

The AVT was taken as the first eigenvariate from the singular value decomposition of a matrix composed of each time series from each voxel within the node-sphere, and the time series of all voxels of the sphere was then element-wise averaged, so as to obtain a single AVT for each node. Based on the AVT data, the network connectivity was then determined by calculating undirected matrices of partial correlations between the nodes, quantifying the unique relationship between each pair of nodes. The calculation of the partial correlations was based on the inverse of the covariance matrix. The partial correlation matrix is a symmetric matrix in which each off-diagonal element is the correlation coefficient between a pair of variables after filtering out the contributions of all other variables included in the dataset. Therefore, the partial correlation between any pair of regions filters out the effects of the other brain regions. Consistent with previous studies, partial correlations were chosen to minimize the impact of indirect dependencies by other brain regions, and to address the problem of complicating the interpretation of the GTNA arising from including multiple redundancies in quantifying inter-nodal functional dependencies when using simple correlation coefficients [48], [49], [50]. Amongst all methods of evaluating functional interdependencies between fMRI time courses in different regions of interest, partial correlations have been found to be amongst the most reliable approaches [51].

In the connectivity matrices, functional connections were defined as valid/existing between pairs of nodes based on the statistical level of significance. We used a threshold of *P*<0.001. Thresholding of the connectivity matrices resulted in binary matrices where existing (valid) connections carried a value of 1 while the absence of a functional connection between network nodes was designated by a value of 0. Self-connections of nodes were not included in the analyses. The resulting thresholded adjacency matrices of partial correlations served as principal input for the GTNA. It has been shown that manipulating the connection density in a network by varying the number of valid network connections can have a noticeable impact on GTNA metrics [52]. Hence, we repeated the GTNA analyses across 5 threshold values (i.e., for each subject, GTNA analyses were calculated based on connectivity matrices thresholded at *P = *0.0001, 0.0005, 0.001, 0.005 and 0.01) to ensure that the statistical results were equivalent across different densities and not critically dependent on the threshold used. Related to the issue of repeating the analyses at different density thresholds is the issue of comparing aspects of network architecture using graph-theory. In order to be able to compare topological features such as the distribution of nodal connectivity degree or the formation and boundaries of different functional communities (functional modules) within a network, it is deemed essential to keep the connection density of the compared networks constant [52]. However, the underlying hypothesis to this study postulates that aging is not only associated with alterations in brain activation but will also modulate functional connectivity in the brain resulting in changes to the number of significant/relevant functional relationships between task relevant areas. In order to capture and quantify this aspect of motor control in aging, we did not enforce the same connection density in our networks for the young and old subjects groups, or the different task conditions. Consistent with this compromise, we report changes in the mean graph-theoretical metrics across each entire network but did not assess specific aspects of network architecture.

In addition to the binary matrices, we also calculated weighted matrices, where for each valid functional connection between a pair of nodes, the value of 1 in the binary matrix was replaced by the value of the partial correlation for this node-pair, with the partial correlation then representing a proxy measure for the weight/strength in connection between this pair of nodes. This approach allowed us to examine connection strength between node-pairs and calculate a mean connection strength for each node defined as the sum of its connection weights (i.e., the sum of partial correlations for valid connections) of a node with other nodes in the network. Subsequently, the mean connection strength of a network was then defined as the mean connection strength across all its network nodes.

### Graph Theoretical Analysis

We applied graph-theoretical network analysis (GTNA) [53], [54] to fMRI data in order to examine and quantify whether the functional connectivity during motor action (i.e., the inter-regional association in the time course of activation) within the cerebral networks for bimanual coordination differs between young and older adults. The examination of complex human brain connectivity using GTNA is a powerful technique to map the relationships between spatially remote neurophysiological events in the brain. Graph-theory is an established mathematical field and has proven a very effective and informative way to explore brain function and human behaviour [53], [54], [55]. Graph-theoretical measures have been applied previously to analyze functional brain connectivity in the context of healthy aging [36], [37], [39], [40], [42]. However, although GTNA offers a broad selection of measures to examine and quantify relationships between activations in different brain regions, previous studies have frequently focused on only a few measures in each case, such as clustering coefficient, network path length, efficiency, small worldness, modularity, or connectivity degree. Furthermore, previous studies (a) commonly used full rather than partial correlations to quantify the association in activation between brain regions, thereby not filtering out the influence of indirect connections between network nodes and (b) applied a template-based a-priori parcellation of the brain to define the network components (nodes), hence including brain regions with little or no task-related activation [36], [37], [39], [40], [42]. A recent study by Smith et al. [51] provides strong indications that, in addition to using partial correlations to quantify unique functional associations between nodes, a data-driven approach by defining networks based only on areas showing clear task-related activation is preferable to template-based approaches in order to minimize confounds and obtain a better picture on functional connectivity within active neural networks. Hence, the present study took advantage of combining the partial-correlation approach with a data-driven network definition.

The main GTNA analyses were conducted using the Brain Connectivity Toolbox [55] (https://sites.google.com/a/brain-connectivity-toolbox.net/bct/Home). We calculated in each network and for each subject the regional (nodal) metrics of the functional network. For each node i of the network, we calculated (see below for measure definitions) the degree K_i_, the local cluster coefficient C_i_, the mean path length L_i_, the local efficiency E_i_, the betweenness centrality b_i_ and the overall connection strength S_i_. For each network, the mean for each of the above GTNA parameters was calculated as the average of each measure across all nodes of a network.

#### Measures of functional connectivity

Connectivity degree is one of the most basic and important measures of network analysis. The degree *K_i_* of a node *i* is defined as the number of connections to that node. Nodes with a high degree are interacting with many other nodes in the network. The degree *K* of a graph is the average of the degrees of all N nodes in the graph G:
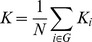



Density can be defined as the fraction of present connections to possible connections.

Connection strength is a measure quantifying how closely network nodes are connected in terms of showing a relationship in their time course of activation. The overall connection strength S_i_ is calculated as:
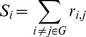
where 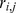 is the partial correlation between the average time series in node i and j.

### Measures of Functional Segregation

Measures of segregation quantify the presence of functionally related, densely interconnected groups of brain regions, known as clusters within the network. The local (nodal) clustering coefficient *C_i_* is defined as the number of existing connections among the node's neighbours divided by all their possible connections:
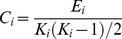
where *E_i_* is the number of existing connections among the node's neighbours. The clustering coefficient of a network is the average of the clustering coefficient of all nodes:



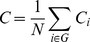
in which *C* quantifies the extent of local connectivity of the network.

Local efficiency E_i_ of a node *i* is related to the clustering coefficient and can be calculated as:
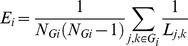
where the subgraph *G_i_* is the set of nodes that are the direct neighbors of the node *i*. This measure reveals how much the network is fault tolerant, showing how efficient the communication is among the first neighbors of the node *i* when it is removed. The mean local efficiency of a graph is defined as the mean of the local efficiency of all the nodes in the graph.

#### Measures of functional integration

Measures of functional integration characterize the ability to rapidly combine specialized information from distributed brain regions and are commonly based on the concept of a path, with shorter paths implying stronger potential for integration.

The mean path length *L_i_* of a node *i* is:
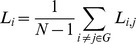
in which *L_i,j_* is the smallest number of edges that must be traversed to make a connection between node *i* and node *j*. The average inverse shortest path length is a related measure known as global efficiency of a network.

#### Measures of centrality

An important measure to assess whether a node has importance for information flow and participates in many shortest paths within a network is *betweenness centrality*. The betweenness centrality *b_i_* of a node *i* is defined as:
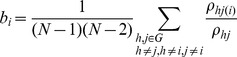
in which 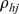 is the number of shortest paths between nodes h and j and 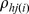 is the number of shortest paths between nodes h and j that pass through node i.

#### Small-world brain connectivity

Global network architecture in terms of the small-worldness was quantified via the normalized clustering coefficient and normalized path length. Previous research has shown that all networks found in biological systems have non-random/non-regular or ‘small-world’ architectures [53], [56]. Small-worldness is a relative measure for a network’s level of functional optimization and deviation from randomness [53], [56], with alterations in this topology commonly representing a decrement in network functionality. Small-world organization incorporates advantages of regular and random networks, preserving optimal levels of connectivity within families of functionally related node-clusters together with short overall communication distances. Hence, small world network character is defined as being more clustered than random networks, yet having approximately the same characteristic path length as random networks [57], that is.
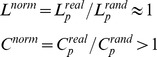
where the 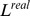 and 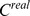
are the mean path length and clustering coefficient of the real network, the 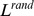 and 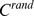 are the equivalent values of matched random networks. In order to obtain the values for the random network parameters in the above equations, we applied a method estimating these random values whilst preserving the principal characteristics of the real network. If the mean vertex degree k of a graph G is defined as k = 2E/N, where E is the number of edges and N is the number of vertices of G, a random graph that is similar to G can be defined with the same mean vertex degree k and number of vertices N as G. Watts and Strogatz [57] defined a reliable estimate of the mean cluster coefficient of a random graph with mean vertex degree k and number of vertices N as C^rand^ = k/N. This estimate can be used to normalize the actual mean cluster coefficient of G for the calculation of ‘small world character’ of a network. Similarly, Watts and Strogatz [57] found an estimate of the mean path length of a random graph L^rand^ = ln(N)/ln(k). Fronczak et al. [58] however showed that this is actually an estimate of the diameter (i.e., the maximal shortest path length). They also derived an analytic solution for the mean path length of a random graph with mean vertex degree k and number of vertices N as L^rand^ = ((ln(N)−γ)/ln(k)) +0.5 (where γ is Euler's constant). This estimate can then be used to normalize the actual mean path length of G with regard to calculation of the ‘small world character’ of a network.

### Statistical Analysis

GTNA measures were analyzed with a 2×2 repeated measures ANOVA with the factors AGE (Old/Young) and PHASE CONDITION (AP/IP). These analyses were conducted for each network separately. Pearson R coefficients were used to examine correlations between GTNA measures and the two key kinematic measures of phase accuracy (error in degrees) and phase stability (SD of relative phase). Correlations below a statistical significance level of *P* = 0.01 were considered significant. All statistical analyses were performed with the Statistica software (StatSoft, Inc).

## Results

### Kinematic Output

Bimanual coordination accuracy and stability did not differ between young and old at any of the relative movement frequencies [30]. For both groups, coordinative performance was significantly decreased during AP versus IP tasks [30]. Movement amplitude was not different between left and right hands and did not significantly differ between age groups [30].

### Differences in Functional Connectivity between Old and Young

#### Effects of age

In both networks, the older group consistently showed the tighter functional connectivity, manifested in a higher mean connectivity degree, connection strength, network density and efficiency, together with shorter mean communication path length between the network nodes and also a lower betweenness centrality (Tables 3 and 4, Figures 2 and 3). In both networks, there were significant effects of AGE for connectivity degree, network density, efficiency, path length, and connection strength (N2 only) (Table 4 & 5). These effects of AGE consistently emerged at stronger levels of statistical significance in the overactivation network N2. While the mean clustering coefficients in network N1 were basically identical between the groups (Table 3), the older group showed higher clustering in network N2, with a significant effect of AGE (Table 4 & 5).

**Table 3:** GTNA metrics for IP and AP in all networks.

	Old group				Young group			
Network	AP		IP		AP		IP	
	Mean	SE	Mean	SE	Mean	SE	Mean	SE
**N1 (** **Table 1** **)**								
Cluster coefficient	**0.29**	0.02	**0.28**	0.01	**0.28**	0.01	**0.28**	0.01
Degree	**4.84**	0.13	**4.71**	0.14	**4.30**	0.15	**4.23**	0.15
Density	**0.24**	0.01	**0.24**	0.01	**0.21**	0.01	**0.21**	0.01
Efficiency	**0.55**	0.01	**0.54**	0.01	**0.51**	0.01	**0.51**	0.01
Path length	**1.97**	0.03	**1.98**	0.04	**2.14**	0.07	**2.16**	0.07
Betweenness	**21.3**	0.70	**21.4**	0.78	**25.0**	1.48	**25.4**	1.45
Connection strength	**1.21**	0.03	**1.18**	0.03	**1.10**	0.04	**1.08**	0.04
**N2 (** **Table 2** **)**								
Cluster coefficient	**0.36**	0.02	**0.33**	0.03	**0.23**	0.03	**0.25**	0.02
Degree	**3.76**	0.12	**3.71**	0.13	**3.11**	0.11	**3.06**	0.11
Density	**0.34**	0.01	**0.34**	0.01	**0.28**	0.01	**0.28**	0.01
Efficiency	**0.58**	0.01	**0.58**	0.01	**0.53**	0.01	**0.53**	0.01
Path length	**1.72**	0.04	**1.74**	0.04	**1.96**	0.06	**1.99**	0.06
Betweenness	**9.67**	0.46	**9.90**	0.49	**12.5**	0.67	**12.9**	0.76
Connection strength	**1.15**	0.04	**1.13**	0.04	**0.98**	0.03	**0.97**	0.02
**Small world network measures**								
**N1**								
Cluster coefficient	**1.27**	0.05	**1.26**	0.04	**1.42**	0.11	**1.45**	0.09
Path length	**0.99**	0.01	**0.99**	0.00	**1.01**	0.01	**1.01**	0.01
**N2**								
Cluster coefficient	**1.16**	0.07	**1.11**	0.10	**0.94**	0.09	**1.00**	0.08
Path length	**0.96**	0.01	**0.96**	0.01	**0.96**	0.01	**0.97**	0.01

**Table 4:** GTNA measures N1−2×2 ANOVA results.

Measure & Factors		0.001		0.0005	0.0001	0.005	0.01
	df	F	*P*	*p*	*p*	*p*	*p*
**Cluster coefficient**							
AGE	1, 30	**0.13**	*0.722*	*0.77*	*0.86*	*0.14*	***0.01***
PHASE	1, 30	**0.68**	*0.423*	*0.94*	*0.45*	*0.53*	*0.22*
AGE×PHASE	1, 30	**1.71**	*0.210*	*0.61*	*0.23*	***0.03***	*0.33*
**Degree**							
AGE	1, 30	**6.03**	***0.027***	*0.13*	***0.03***	***0.02***	***0.04***
PHASE	1, 30	**12.3**	***0.003***	***0.003***	*0.50*	*0.29*	*0.43*
AGE×PHASE	1, 30	**2.36**	*0.145*	*0.10*	*0.64*	***0.04***	*0.59*
**Density**							
AGE	1, 30	**6.03**	***0.027***	*0.13*	***0.03***	***0.02***	***0.04***
PHASE	1, 30	**12.3**	***0.003***	***0.003***	*0.50*	*0.29*	*0.43*
AGE×PHASE	1, 30	**2.36**	*0.145*	*0.10*	*0.64*	***0.04***	*0.59*
**Efficiency**							
AGE	1, 30	**6.29**	***0.024***	*0.36*	***0.07***	***0.02***	***0.04***
PHASE	1, 30	**9.18**	***0.008***	*0.13*	*0.42*	*0.30*	*0.28*
AGE×PHASE	1, 30	**0.81**	*0.384*	***0.03***	***0.04***	***0.02***	*0.56*
**Path length**							
AGE	1, 30	**6.46**	***0.023***	***0.05***	***0.03***	***0.02***	***0.05***
PHASE	1, 30	**2.05**	*0.173*	*0.71*	*0.50*	*0.24*	*0.23*
AGE×PHASE	1, 30	**0.11**	*0.747*	*0.33*	*0.29*	***0.02***	*0.65*
**Betweenness**							
AGE	1, 30	**6.74**	***0.020***	***0.02***	***0.04***	***0.02***	***0.05***
PHASE	1, 30	**1.01**	*0.331*	*0.47*	*0.99*	*0.24*	*0.23*
AGE×PHASE	1, 30	**0.29**	*0.599*	*0.31*	*0.37*	*0.02*	*0.65*
**Connection strength**							
AGE	1, 30	**4.05**	*0.063*	*0.18*	*0.07*	*0.05*	*0.06*
PHASE	1, 30	**25.5**	***0.0001***	***0.0001***	***0.04***	***0.01***	***0.004***
AGE×PHASE	1, 30	**4.51**	*0.051*	*0.20*	*0.50*	*0.17*	*0.44*
**Small World Network measures**							
**SWN Cluster coefficient**							
AGE	1, 30	**2.31**	*0.150*	*0.49*	*0.20*	*0.61*	*0.26*
SESSION	1, 30	**0.12**	*0.732*	*0.40*	*0.44*	*0.11*	*0.11*
AGE×SESSION	1, 30	**0.34**	*0.566*	*0.68*	*0.26*	***0.01***	*0.35*
**SWN Path length**							
AGE	1, 30	**3.08**	*0.100*	*0.65*	*0.30*	*0.49*	*0.94*
SESSION	1, 30	**1.04**	*0.324*	*0.65*	*0.68*	*0.35*	*0.20*
AGE×SESSION	1, 30	**0.68**	*0.422*	***0.03***	*0.26*	***0.04***	*0.77*

**Table 5:** GTNA measures N2−2×2 ANOVA results.

Measure & Factors		0.001		0.0005	0.0001	0.005	0.01
	df	F	*P*	*p*	*p*	*p*	*p*
**Cluster coefficient**							
AGE	1, 30	**7.52**	***0.015***	***0.048***	***0.04***	*0.06*	***0.05***
PHASE	1, 30	**0.27**	*0.613*	*0.69*	*0.89*	*0.97*	*1.00*
AGE×PHASE	1, 30	**2.56**	*0.130*	*0.34*	*0.17*	*0.81*	*0.45*
**Degree**							
AGE	1, 30	**13.7**	***0.002***	***0.002***	***0.004***	***0.002***	***0.01***
PHASE	1, 30	**4.75**	***0.046***	*0.16*	*0.50*	***0.02***	*0.06*
AGE×PHASE	1, 30	**0.00**	*1.000*	*0.87*	*1.00*	*0.40*	***0.03***
**Density**							
AGE	1, 30	**13.7**	***0.002***	***0.002***	***0.004***	***0.002***	***0.01***
PHASE	1, 30	**4.75**	***0.046***	*0.16*	*0.50*	***0.02***	*0.06*
AGE×PHASE	1, 30	**0.00**	*1.000*	*0.87*	*1.00*	*0.40*	***0.03***
**Efficiency**							
AGE	1, 30	**13.7**	***0.002***	***0.02***	***0.006***	***0.001***	***0.006***
PHASE	1, 30	**8.04**	***0.013***	*0.14*	*0.22*	***0.02***	*0.13*
AGE×PHASE	1, 30	**0.02**	*0.883*	*0.56*	*0.82*	*0.41*	*0.08*
**Path length**							
AGE	1, 30	**11.2**	***0.004***	***0.004***	***0.01***	***0.002***	***0.004***
PHASE	1, 30	**8.39**	***0.011***	*0.54*	*0.21*	***0.04***	*0.33*
AGE×PHASE	1, 30	**0.15**	*0.706*	*0.71*	*0.71*	*0.38*	*0.26*
**Betweenness**							
AGE	1, 30	**11.2**	***0.004***	***0.03***	***0.01***	***0.002***	***0.004***
PHASE	1, 30	**8.39**	***0.011***	*0.99*	*0.21*	***0.04***	*0.33*
AGE×PHASE	1, 30	**0.15**	*0.706*	*0.87*	*0.71*	*0.38*	*0.26*
**Connection strength**							
AGE	1, 30	**13.2**	***0.002***	***0.004***	***0.004***	***0.001***	***0.003***
PHASE	1, 30	**12.8**	***0.003***	***0.05***	*0.08*	***0.003***	***0.004***
AGE×PHASE	1, 30	**0.00**	*0.986*	*0.91*	*0.99*	*0.39*	***0.03***
**Small World Network measures**							
**SWN Cluster coefficient**							
AGE	1, 30	**1.79**	*0.201*	*0.18*	*0.27*	*0.51*	*0.51*
SESSION	1, 30	**0.00**	*0.954*	*0.82*	*0.72*	*0.35*	*0.47*
AGE×SESSION	1, 30	**1.87**	*0.191*	*0.50*	*0.17*	*0.89*	*0.78*
**SWN Path length**							
AGE	1, 30	**0.17**	*0.687*	*0.11*	*0.88*	*0.58*	*0.32*
SESSION	1, 30	**0.40**	*0.538*	***0.03***	*0.17*	*0.44*	*0.87*
AGE×SESSION	1, 30	**0.10**	*0.751*	*0.05*	*0.89*	*0.98*	*0.45*

#### Effects of task difficulty

Compared to the easier IP coordination mode, the AP mode was associated with an increased functional connectivity, with a significant effect for PHASE in all networks for the measures connectivity degree, network density, efficiency, path length (N2 only), and connection strength (Table 4 & 5). However, there was no AGE X PHASE interaction at the examination threshold of *P = *0.001 (Table 4 & 5).

The comparison of the functionality measures obtained for the participants’ brain networks with equivalent parameters derived from random networks indicated a topology largely consistent with a ‘small world’ network (SWN) character in both groups (Table 3). No statistically significant group differences were observed regarding this ‘small world’ topology (Table 4 & 5).

#### Interhemispheric connectivity

Previous studies have demonstrated an increased bihemispheric activation in older adults compared to a more lateralized processing in younger individuals [59], [60]. Based on this notion of a changing balance of communication between the hemispheres during motor processing in the aging brain, we conducted an exploratory sub-analysis to examine changes in overall strength of interhemispheric connectivity in our groups via a 2×2 AGE×PHASE ANOVA. This analysis included only connections between network nodes in different hemispheres (i.e., the functional connections ‘travel’ between the hemispheres). In network N1 the older group had the numerically stronger interhemispheric connectivity for the AP task (mean connection strength of interhemispheric connections ± SE: 0.62±0.02 vs. young: 0.56±0.03) and the IP task (old 0.59±0.02 vs. young 0.56±0.03) but these differences were too small to result in a significant effect of AGE [F(1, 15) = 1.73, *P* = 0.21]. However, the effect of PHASE was significant [F(1, 15) = 14.65, *P = *0.002], and there was a significant AGE×PHASE interaction [F(1, 15) = 7.88, *P* = 0.013]. Post hoc tests using Tukey correction showed that interhemispheric connectivity was significantly higher in the AP vs. the IP tasks for the older group (*P* = 0.001), whereas no such difference was present for the young participants (*P* = 0.78).

In N2, the findings were similar, with a significant effect of PHASE [F(1, 15) = 18.5, *P = *0.0006] and a numerically higher interhemispheric connectivity for the older group in the AP task (mean connection strength of interhemispheric connections ± SE: 0.37±0.01 vs. young: 0.36±0.01) and the IP task (old 0.36±0.02 vs. young 0.35±0.01). However, there was neither a significant effect of AGE [F(1, 15) = 0.43, *P* = 0.52] nor a AGE×PHASE interaction [F(1, 15) = 0.02, *P* = 0.91].

### Correlations between GTNA Measures and Kinematics

The exploratory analysis of correlations between the GTNA measures and the kinematic parameters showed that, after correcting for number of correlations evaluated, there was little association between the measures of functional connectivity and the two key kinematic measures of phase accuracy (error in degrees) and phase stability (SD of relative phase) [30]. In the analyses based on combining activations across different movement frequencies, significant correlations between GTNA measures and the means of the kinematic performance measures across the 4 movement frequencies were largely absent in both networks, the exception being correlations of the AP mean phase stability with SWN path length (R = 0.64, *P* = 0.007) and with the mean cluster coefficient (R = 0.62, *P* = 0.009) in the N1 network of the young group, whereby poorer stability in the AP task (i.e., a higher SD of relative phase) was linked to longer communication path lengths but also to increased local clustering patterns.

In the primary fMRI analyses that established the brain activations underlying the present neural networks [30], two regions (SMA and the secondary somatosensory area) of the overactivation network N2 had demonstrated a positive relationship between activation and motor performance in elderly subjects during the AP task. However, the examination of associations between task performance and GTNA measures in these two areas showed only a limited number of significant correlations between increased functional connectivity and improved task performance (smaller errors) in the older group. For the SMA, this included correlations of AP phase accuracy with efficiency (R = −0.63, *P*<0.01) and connection strength (R = −0.69, *P*<0.01), as well as correlations of AP phase stability with connection strength (R = −0.70, *P*<0.01). In the secondary somatosensory area, no correlations with kinematics were present. In the young group, no significant correlations were present between the GTNA measures and the kinematic output in these two ROIs.

### GTNA Analyses at Different Connectivity Thresholds

**Figure 4:**
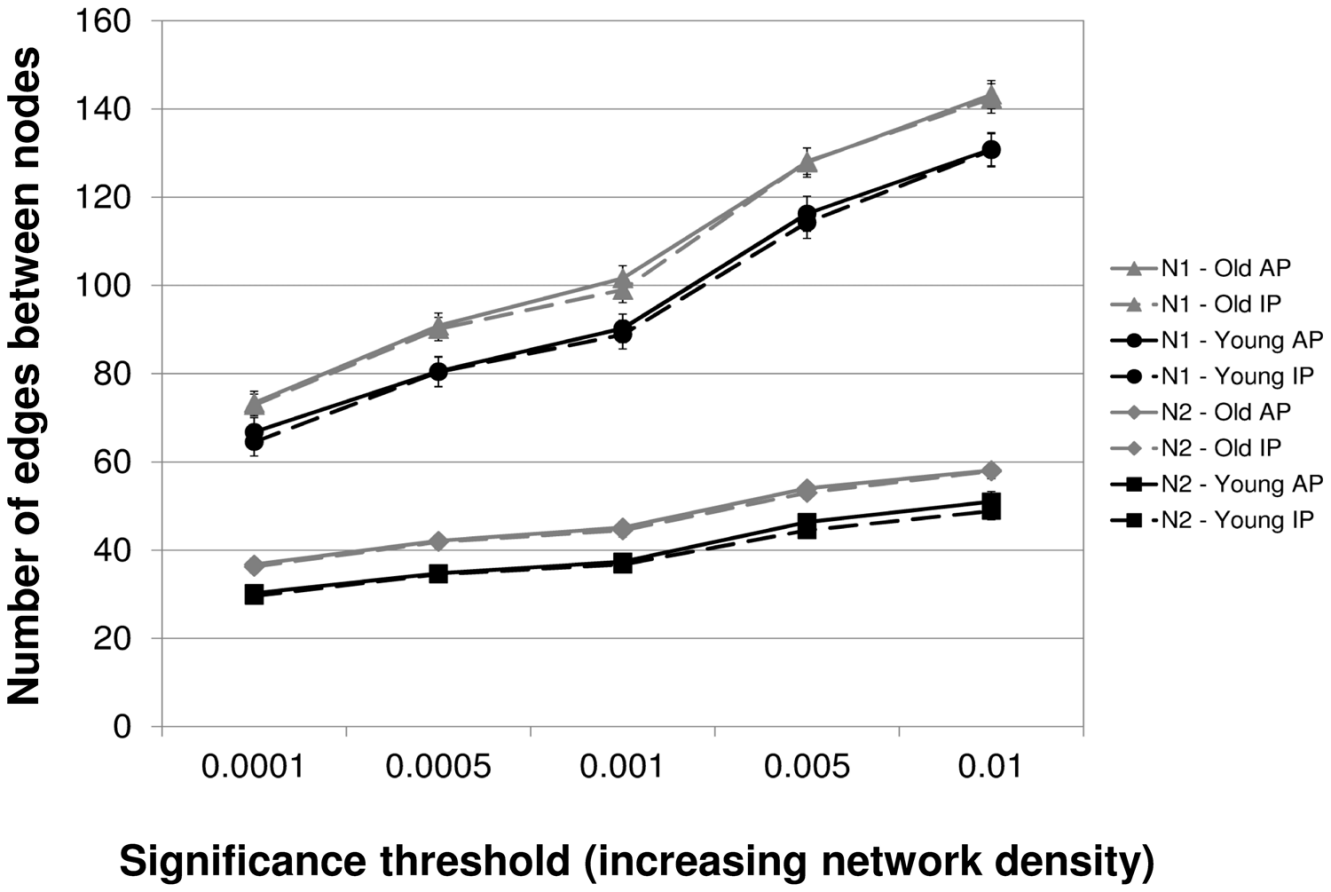
Number of edges between network nodes at different connectivity (network density) thresholds. Shown are the number of valid functional connections (edges) based on significant partial correlations between node-pairs in networks N1 and N2. Consistent with the notion that fewer connections will be considered as ‘existing’ at lower compared to higher *P*-level thresholds, both networks showed a steady increase in the number of existing edges across the 5 different connectivity thresholds.

We calculated the connectivity matrices at different thresholds (*P* = 0.0001; *P* = 0.0005; *P* = 0.001; *P* = 0.005; *P* = 0.01) to ensure that the results were not critically dependent on the threshold used. The statistical results were equivalent across network densities and the secondary thresholds showed no consistent statistical trends that were critically different to that of the examination threshold of *P* = 0.001. For all connectivity thresholds, the numbers of surviving edges between the network nodes are shown in Figure 4.

## Discussion

This study assessed the functional connectivity in the cerebral networks for bimanual coordination in older adults by applying graph-theoretical network analysis. The study evaluated functional connectivity aspects in the elderly and is the first to show that bimanual coordination in older adults occurs concomitantly with (a) an increased functional network connectivity between brain areas also activated in young adults, and (b) an increased functional connectivity in the overactivation network specific to the older age group. This effect was independent of ‘phase’ complexity, although, compared to the IP condition, the more difficult AP mode was associated with an increased network connectivity in both age groups. Importantly, the current findings demonstrate that the age-related overactivation seen in some areas (N2) does not appear to be associated with a decreased network functionality in the ‘common’ network (i.e., the task-related motor networks employed by both old and young adults, N1).

### Increased Functional Connectivity in the Elderly – does Higher Mean Better?

Our finding of an increased functional connectivity applied consistently across the vast majority of GTNA measures, confirming that this effect does not only focus on sub-domains of functional connectivity but appears to relate homogeneously to the entire functionality spectrum assessed in the present networks. Importantly, this increased functional connectivity was not carried to a critical extent by an increased connection strength in interhemispheric links that one might have expected based on previous reports of a decreased lateralization of motor processing in older adults [59], [60].

The current findings also show this age effect to be independent of the ‘phase’-difficulty of the movement task when considering the entirety of the networks as, in both groups, the more difficult AP condition was consistently associated with a tighter functional connectivity compared to the simpler and more intrinsically executable IP condition. A priori, it would have been feasible to assume that this disparity gradient in the quality of functional connectivity between AP and IP condition might have been steeper in the elderly due to their brain having to work harder to maintain AP motor output. Our analyses showed that this was the case when focussing on the strength of interhemispheric connections. Here, the older group showed a tighter interhemispheric connectivity than the young in the more difficult AP condition. Whilst this finding seems consistent with the notion of a decreased lateralisation in older adults, our results suggest that a tighter interhemispheric connection strength is dependent on task difficulty and that the effect of AGE may be more specifically reflected only in more demanding coordination modes.

A crucial point that needs to be considered in the interpretation of the current findings is the question of whether the increased functional connectivity of the elderly group indeed represents a ‘better’ functionality. When assessing functional connectivity in a network, it seems intuitive that any improvements which promote connections between network components (increased clustering, higher network density, more connections of the network node, and stronger synchronization in activation between network components), together with quicker information transfer (shorter communication path lengths and decreased betweenness centrality) are of benefit to network performance. In this context, it is pertinent to mention that our finding of a reduced betweenness centrality in the older subjects is consistent with them having shorter mean path lengths between network nodes compared to the young group. Shorter path lengths are a manifestation of a more direct connection between network nodes, resulting in less nodes participating in shortest paths between other network nodes. This is likely to directly affect the betweenness centrality as this measure quantifies the number of shortest paths (connecting other nodes of the network) that travel through each node. As a result, the betweenness of the network will be reduced as a whole.

In over-connected functional systems, however, the balance between network performance and the required connectivity architecture can become distorted so that system performance decreases as a result [53]. The apparent advantage in functional topology of the elderly subjects has to be interpreted in the light of the unchanged small world character of their networks compared to the younger controls. In efficient networks there is an optimal balance between the performance output and the best level of connectivity to deliver that output. In many biological systems this optimal configuration has been summarized in the term ‘small worldness’ [53]. Small-world organization combines, and finds an optimal compromise between, the short overall path lengths of random networks and the high-level of clustering of regular networks. As such it combines high levels of local clustering among nodes of a network and short paths that globally link all nodes of the network. The preserved small world character of the elderly indicates that the apparently ‘better’ functional connectivity in their networks was established within the intact framework of a small world system comparable to that of the young. Considered together with the fact that the changes in functional connectivity seen in the older adults are of a nature that homogeneously promotes better network function, the finding of a preserved small world character supports the interpretation that the GNTA measures of the older group are indeed indicative of a more favourable functional connectivity of this group.

### Increased Functional Connectivity in the Elderly in Addition to Overactivation

The compensation hypothesis for the additional neural recruitment (or overactivation) of the elderly [24], [59] assumes that, in order to maintain performance equivalent to the young in both the cognitive domain and a motor context, the overactivation areas compensate for processing deficiencies or a declining computational efficiency in areas more commonly used at a younger age. Based on this assumption, age-equivalent performance of the elderly is essentially due to benefitting from this additional neural recruitment. The old group in the present study also showed such overactivation, demonstrating for the first time that bimanual movements require greater neural resources for old adults in order to match the level of performance seen in younger subjects [30]. While it may not be surprising that the GTNA measures for the overactivation network N2 indeed assigned an increased functional connectivity to this network in the older group, our present analysis shows that the functional connectivity in the common network N1 was also higher in the elderly. This suggests that the greater neural activation of the older group in the secondary network N2 [30] was not induced by a poorer functionality in the common network N1. In turn, this indicates that the ability of the elderly to maintain similar levels of bimanual coordination compared to the young was not solely due to benefitting from overactivation but also to a tightening of functional relationships between brain regions also recruited by younger subjects during task performance. One might argue that the increased functional connectivity of the elderly was facilitated by the stronger activation of the older group in the assessed networks, whereby homogeneously stronger activations across all network regions might drive stronger correlations between these areas. However, while this argument might apply to the overactivation network N2, it cannot account for the better functional connectivity in the common network N1, which, in turn, suggests that the present findings are indeed indicative of an increased functional connectivity in the elderly group.

The increased functional connectivity in the older adults suggests more tightly synchronized brain activation patterns that apply during motor coordination. This finding is consistent with previous notions of changes in brain activation in response to a gradually failing neurobiological substrate during advanced aging [59], [60]. Decrements in neurobiological quality promote the brain becoming less versatile in coping with diversity in the activity levels among its network nodes, which may lead a simplified functional architecture characterized by increased functional coherence between network areas as seen in our present results. It follows that the activity levels across multiple network nodes will show a higher level of synchronization compared to younger adults, concomitantly with successful bimanual coordination. This interpretation is consistent with previous research having shown that the brain of older adults indeed exhibits a significantly lower variability in task-related activations compared to younger controls, and that the aging brain has a poorer ability of exploring different functional states across brain regions [61]. Our present results indicate that this likely applies also during complex bimanual motor control.

### Correlations between Functional Connectivity and Motor Performance

Our analyses showed that, when assessing the relationship between task performance and the mean GTNA metrics calculated across all network nodes of N1 and N2 respectively, there was limited association between the level of functional network connectivity and motor performance in the older group. Two significant correlations between GTNA measures and coordinative accuracy were observed in the N1 network of the young group, and there exclusively for the more complex AP condition. Our more focused correlation analysis examined the relationship between functional network connectivity and motor performance in two areas that showed a positive relationship between BOLD activation and motor performance in elderly subjects during the AP task (the SMA and SII region) [30]. However, only in the SMA a stronger link with the activation levels in other network nodes (connection strength), and shorter communication pathways with other nodes (increased efficiency) were associated with better motor performance (reduced error). While these links between functional connectivity and motor performance in the more difficult AP task are encouraging, it has to be acknowledged that the number of links between GTNA measures and motor performance in the present study was limited, which may have been due to only assessing the means of the GTNA measures taken across the entire networks in each subject.

### Study Limitations and Considerations for Interpreting the Current Findings

The present study combined a partial-correlation approach to quantify functional connectivity with a data-driven network definition. This approach was chosen as full correlations have been criticized for not filtering out the influence of indirect connections between network nodes and template-based a-priori parcellation of the brain to define the network nodes has the caveat of including brain regions with little or no task-related activation. A recent study by Smith et al. [51] provides strong indications that, in addition to using partial correlations to quantify unique functional associations between nodes, a data-driven approach by defining networks based only on areas showing clear task-related activation is preferable to template-based approaches in order to minimize confounds and obtain a better picture on functional connectivity within neural networks. Using partial correlations allowed us to focus on the unique variance shared by any pair of network nodes while removing all effects causing spurious correlations between two regions as a result of a correlation with a common factor. This makes the use of partial correlations very promising even in task based fMRI. Amplitudes of partial correlations are typically low but by using a statistical criterion we ensured that we only assess functional connectivity between regions sharing unique variance. However, it has to be acknowledged that the best methodological approach to examine functional connectivity is an area of ongoing debate.

It is important to note that defining network nodes based on task-related activation has the potential problem of being based on a group analysis rather than subject-specific analyses, and, at least in our context, on incorporation of general linear models (GLMs) rather than models that allow for nonlinear effects. However, the application of the GLMs, and the inference of the group results being representative of brain activations in the individual, is established practice in fMRI analysis and was successfully applied for the primary imaging analysis of the present data [30]. Using the same GLMs to assess functional connectivity between time courses of activation in our subjects presented itself as the natural progression to the primary imaging analysis and ensured that the examination of functional connectivity was established within, and relates to, the framework of cerebral activations identified by the GLM method. Despite a good argument to only focus on areas with task-related activation for the purposes of task-related changes in functional connectivity, it has to be considered that a network definition based on a limited number of task-related ROIs focuses on partial brain rather than whole brain examination, and that this method may therefore omit potentially relevant activation in other brain areas. However, the method of task-related ROIs ensures that the identified connectivity can be comfortably linked to the task in question and minimizes the risk of including spurious contributions of potentially ‘random’ brain activity.

Our main analyses showed consistent increases in functional connectivity in the elderly that manifested across multiple graph-theoretical metrics, showing that this increase in mean functional connectivity could be captured across multiple (albeit inter-related) sub-domains of functional connectivity. However, it has to be emphasized that in the present analysis the number of network connections was not held constant between groups and conditions. A disadvantage of having a different number of connections (i.e., not limiting the network connections to a constant number in the young and old groups) in our networks is that we cannot make inferences about specific topological aspects of the networks [52]. Hence, the present findings do not prove that the increased functional connectivity between young and old is due to significant changes in network topology but might have been carried substantially by an increase in number of network connections.

It also should be noted that the sample size of this study was limited. However, this present sample size was sufficient to adequately detect and describe fMRI activations related to the performance of the study task [30]. It follows that the underlying brain activations were likely sufficiently strong to meaningfully examine functional correlations between the brain areas activated in the task.

In interpreting our current findings, it is important to emphasize that the present findings should not automatically be seen as direct support of the compensation hypothesis for the additional neural recruitment of the elderly [24], [59]. This hypothesis only refers to age-related higher levels of brain activation in older adults, which is, in turn, also expected to show correlations with motor performance. However, this hypothesis does not make any statements towards connectivity differences between young and old. Whilst age-related additional recruitment and a closer functional network connectivity might be concomitantly occurring coping mechanisms of the aging brain, caution may be advised in assuming that these two processes are causally impacting on each other.

### Relevance of the Present Findings

There are previous reports that mirror our finding of a higher functional connectivity in the elderly, such as a higher number of connector nodes in the elderly when assessing network modularity across the entire brain at resting state [37] and a reduced regional centrality in frontal areas during memory encoding and recognition [36]. Our results showed an increased network efficiency for older subjects, a finding consistent with the findings of the study of Park and colleagues, the only previous study that has examined aspects of functional connectivity in a task-related motor context [42]. They found that their key measure of network efficiency also increased with age when focusing on connectivity in parietal-occipital-cerebellar networks, although it has to be emphasized that this finding in Park’s study was present in this particular sub-network and did not apply globally thus limiting consistency of our findings with those of Park et al. [42]. Our findings of a higher task-related functional connectivity, however, are consistent with previous findings of higher resting state connectivity between neuroanatomical regions crucial for motor coordination [41]. However, it has to be acknowledged that some studies have found that aging decreases the functional connectivity in the context of cognitive function and resting state activity of some motor-related brain structures [36], [37], [38], [39], [40].

In our present analysis, we add to those previous findings by specifically examining aspects of functional connectivity related to age-related overactivation, which is one of the most central research questions in the field of cognitive neuroscience of aging. Our present study comprised a more comprehensive selection of GNTA measures and expands previous findings by showing that adequate sensorimotor performance in older adults occurs concomitantly with an increased functional network connectivity between (a) brain areas also activated in young adults, and (b) brain areas showing overactivation specifically in older age groups. The present analysis was further able to demonstrate that the overactivation seen in older adults does not seem to be associated with the incidence of a decreased network functionality in the ‘common’ brain network (i.e., the task-related cerebral areas employed similarly by old and young adults).

In summary, the current findings indicate that in the context of task-driven bimanual motor control in the elderly, additional neural recruitment may not be the only mechanism of the aging brain to preserve motor output. Our findings indicate that the aging brain adapts and increases functional connectivity among the nodes of the brain network, presumably to cope with age-related structural and biochemical changes.
